# Joint Associations of Accelerometer-Derived Intensity Gradient and Diet Quality with Frailty Among Rural Chinese Older Adults

**DOI:** 10.3390/nu18081185

**Published:** 2026-04-09

**Authors:** Ke Chen, Yating Liu, Ming Li, Meng Zhao, Kunli Wang, Ziwen Pan, Si Chen, Kefang Wang

**Affiliations:** 1 School of Nursing and Rehabilitation, Shandong University, Jinan 250012, China; 2 Department of Neurosurgery, Qilu Hospital, Cheeloo College of Medicine and Institute of Brain and Brain-Inspired Science, Shandong University, Jinan 250012, China

**Keywords:** frailty, rural older adults, diet quality, intensity gradient, average acceleration

## Abstract

Background/Objectives: Frailty is common among rural Chinese older adults despite relatively high daily physical activity, a phenomenon known as the “rural frailty paradox.” Conventional moderate-to-vigorous physical activity (MVPA) metrics rely on absolute cut-points and are often highly correlated with activity volume, limiting their ability to distinguish the roles of activity volume and activity intensity distribution. We therefore applied a cut-point-free accelerometer approach using average acceleration (AvAcc) and intensity gradient (IG) to distinguish activity volume from activity intensity distribution and to examine whether activity intensity distribution, together with diet quality, could help explain the rural frailty paradox beyond total activity volume alone. Methods: In this cross-sectional analysis of the Healthy Aging and Lifestyle Enhancement study, 1203 rural older adults were included. Physical activity (PA) was objectively measured using triaxial accelerometers to derive AvAcc and the IG. Diet quality was assessed using the China Prime Diet Quality Score (CPDQS), and frailty was assessed using the Fried frailty phenotype adapted for rural Chinese older adults. Multiple linear regression, joint effect models, and restricted cubic spline analyses were conducted after adjustment for age, sex, chronic disease status, total energy intake, and related covariates. Results: In mutually adjusted models, higher IG and CPDQS were independently associated with lower frailty scores, whereas AvAcc was not. In the fully adjusted model, IG (β = −0.14, *p* < 0.001) and CPDQS (β = −0.10, *p* < 0.001) were inversely associated with frailty score, while AvAcc showed no significant association (*p* = 0.665). In joint analyses, compared with the low-IG/low-CPDQS group, participants with high IG/high CPDQS had the lowest frailty scores (β = −0.28, *p* < 0.001), followed by those with low IG/high CPDQS (β = −0.20, *p* = 0.002). Restricted cubic spline analyses indicated a non-linear association between IG and frailty and an approximately linear inverse association for CPDQS. Conclusions: These findings suggest that, among rural older adults, frailty may be more strongly associated with activity intensity distribution than with total activity volume alone. Together with diet quality, this may help explain the rural frailty paradox.

## 1. Introduction

Frailty, a geriatric syndrome defined by diminished multisystem physiological reserves and reduced stress resilience [1], is a critical independent predictor of adverse outcomes, including disability and all-cause mortality. The prevalence of frailty among adults aged 60 years and older in China has been reported to be 10.1% [2]. Due to limited healthcare access and inferior chronic disease management, rural seniors face higher frailty risk and an urgent need for targeted interventions. While physical activity (PA) is widely considered an important modifiable factor in frailty research [3], a paradoxical phenomenon persists in rural populations: high activity volume [4] coexists with an elevated prevalence of frailty [5]. This contradiction suggests that evaluating PA solely through activity volume may overlook important aspects of the association between PA and frailty, as the relationship of PA with muscle and function may differ according to activity intensity and nutritional context [6,7].

PA intensity has been proposed as a key driver of frailty dynamics; however, evidence based on moderate-to-vigorous PA (MVPA) remains inconsistent. While some studies report an inverse association between objectively measured MVPA and frailty [8], others suggest that meeting MVPA recommendations is not necessarily associated with frailty prevention or lower frailty prevalence [9]. These discrepancies may stem from the inherent methodological limitations of the “cut-point” approach in older and frail populations [10]. First, absolute intensity cut-points fail to account for the relative effort of frail individuals, leading to systematic misclassification and underestimation of physiological strain [11]. Second, MVPA reduces the 24 h movement profile to a binary accumulation of time above a threshold, disregarding the continuous intensity distribution across the day [10]. This is particularly problematic in frail populations whose activity may predominantly occur at lower intensities that fall below traditional thresholds but are still clinically meaningful. Third, conventional MVPA metrics are often strongly correlated with total activity volume (e.g., average acceleration, AvAcc) [12]. This makes mutual adjustment statistically difficult and limits the ability to determine whether frailty is more closely related to activity volume or to activity intensity distribution. To address these limitations, emerging research advocates for a “cut-point-free” approach using two complementary metrics: AvAcc and the intensity gradient (IG). While AvAcc represents activity volume, IG serves as a comprehensive descriptor of activity intensity distribution across the entire 24 h spectrum. Unlike MVPA, IG does not target a specific intensity category; instead, it captures how activity time is distributed across all intensity levels—from light to vigorous—by reflecting the rate of decline in time as intensity increases. Compared with conventional cut-point-based metrics, this approach avoids reliance on arbitrary absolute thresholds, captures the full activity intensity distribution across the day, and allows activity volume and intensity distribution to be examined simultaneously [12]. Accordingly, the combined use of AvAcc and IG may provide a more nuanced understanding of how different dimensions of physical activity relate to frailty.

From a nutritional perspective, diet quality provides the biochemical foundation for muscle protein synthesis and the maintenance of metabolic homeostasis. Prior evidence linking nutrition to frailty has largely focused on urban community-dwelling populations [13] and Mediterranean-style dietary patterns [14,15], which may not be generalizable to rural China due to substantial differences in food accessibility and lifestyle. Moreover, many nutritional studies treat PA as a coarse, self-reported covariate, which may introduce substantial residual confounding—particularly in rural settings where activity patterns are complex and labor-related.

Importantly, nutrition and PA are biologically interdependent. Diet quality provides substrates for anabolic processes, whereas activity intensity distribution (captured by IG) may act as a mechanical signal initiating anabolic pathways. However, few studies have examined whether activity intensity distribution, independent of total activity volume, is associated with frailty, and to our knowledge, no study has jointly investigated cut-point-free PA metrics and diet quality in relation to frailty among rural Chinese older adults. This gap is particularly relevant to the rural frailty paradox, in which relatively high habitual activity coexists with elevated frailty risk. Therefore, this study aimed to examine the independent and joint associations of AvAcc, IG, and CPDQS with frailty among rural older adults, and to determine whether activity intensity distribution and diet quality provide additional insight into the rural frailty paradox beyond activity volume alone.

## 2. Materials and Methods

### 2.1. Study Design and Sample

Participants in this study were drawn from the baseline survey of the “Healthy Aging and Lifestyle Enhancement” project. This community-based initiative aims to examine the associations between lifestyle, physical activity, and health outcomes among rural older adults through multidimensional data collection, thereby providing a scientific basis for promoting healthy aging. Data were collected by trained assessors with professional backgrounds in geriatric nursing and rehabilitation, all of whom completed a standardized training protocol. From April to August 2024, participants were recruited from townships in Dezhou, Shandong Province. A total of 1906 older adults were initially recruited. The inclusion criteria were as follows: (1) aged 65 years or older and residence in rural areas; (2) ability to communicate effectively and maintain independence in activities of daily living; and (3) provision of written informed consent for voluntary participation. Individuals were excluded if they had acute medical events, terminal illnesses, or severe cognitive impairment. Participants with missing data on accelerometry, frailty, or relevant covariates were also excluded. Consequently, a final sample of 1203 participants was included in the statistical analysis.

### 2.2. Assessment of Frailty: The Fried Frailty Phenotype

The frailty status of participants was assessed using the Fried Frailty Phenotype, adapted and validated for the Chinese population by the Geriatrics Branch of the Chinese Medical Association [16]. Frailty was evaluated based on the following five components, with each fulfilled criterion assigned one point:

(1) Exhaustion: Participants were classified as exhausted (1 point) if they reported experiencing either of the following conditions for three or more days during the past week: (a) “I felt that everything I did was an effort,” or (b) “I could not get going.”
(2) Weakness: Grip strength was measured using the dominant hand, and the maximum value from two trials was recorded. One point was assigned if grip strength fell below the sex- and body mass index (BMI)-specific cutoffs. For males, the thresholds were ≤29 kgf for BMI ≤ 24.0 kg/m^2^, ≤30 kgf for BMI 24.1–26.0 kg/m^2^ or 26.1–28.0 kg/m^2^, and ≤32 kgf for BMI > 28.0 kg/m^2^. For females, the thresholds were ≤17 kgf for BMI ≤ 23.0 kg/m^2^, ≤17.3 kgf for BMI 23.1–26.0 kg/m^2^, ≤18 kgf for BMI 26.1–29.0 kg/m^2^, and ≤21 kgf for BMI > 29.0 kg/m^2^.
(3) Slowness: Walking speed was determined by the time required to walk a 4.57 m distance at a usual pace. Slowness (1 point) was defined according to sex- and height-stratified cutoffs: For males, slowness was defined as a walking time ≥7 s for height ≤173 cm or ≥6 s for height >173 cm. For females, slowness was defined as ≥7 s for height ≤159 cm or ≥6 s for height >159 cm.
(4) Physical Inactivity: Energy expenditure was estimated using the International Physical Activity Questionnaire-Short Form. One point was assigned if the weekly physical activity fell below the established thresholds: <383 kcal/week for males and <270 kcal/week for females.
(5) Unintentional Weight Loss: Participants were assigned 1 point if they reported an unintentional weight loss of more than 4.5 kg or >5% of their total body weight within the previous year.

The total frailty score ranged from 0 to 5. Based on the aggregate score, participants were categorized as robust (0), pre-frail (1–2), or frail (≥3).

### 2.3. Measurement of Physical Activity

**Figure 1:**
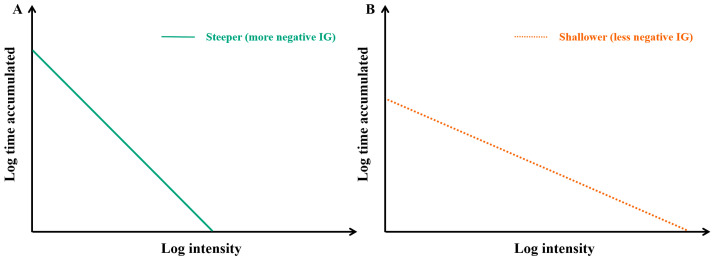
Conceptual illustration of two examples of the intensity gradient (IG). Notes: Panel (**A**) illustrates a steeper (more negative) intensity gradient, indicating that accumulated time is concentrated at lower intensities; Panel (**B**) illustrates a shallower (less negative) gradient, indicating a more favorable activity intensity distribution, with a relatively greater proportion of activity accumulated at higher intensities.

PA was objectively measured using the ActiGraph wGT3X-BT triaxial accelerometer (ActiGraph LLC, Pensacola, FL, USA), which has been widely used in studies of adults and older adults [17]. Participants were instructed to wear the device on their non-dominant wrist 24 h/day for seven consecutive days, removing it only for bathing or water-based activities. The sampling frequency was initialized at 80 Hz. Raw acceleration data were downloaded using ActiLife software (version 6.13.4) and processed in R (version 4.5.1) using the open-source GGIR package. The magnitude of dynamic acceleration was calculated using the Euclidean Norm Minus One method in 5 s epochs and expressed in milli-gravitational units (mg). PA metrics were extracted over a 24 h cycle (0:00 to 24:00) and averaged across all valid wear days. Non-wear time was identified using the GGIR algorithm based on acceleration variability within moving windows. Specifically, 60 min windows were moved in 15 min increments, and a window was classified as non-wear if at least two of the three axes showed a standard deviation <3 mg or a value range <50 mg. Participants were excluded from the analysis if they met any of the following criteria: failure of the auto-calibration (post-calibration error ≥ 0.01 g), fewer than three valid wear days (defined as ≥16 h of wear time per day), or a lack of wear data for any 15 min window within the 24 h cycle. The primary accelerometer-derived PA metrics included AvAcc and IG [11,12]. AvAcc reflects activity volume, with higher values indicating greater overall activity volume. A change of approximately 1 mg in AvAcc has been proposed as a minimum clinically important difference in inactive adults [18], which may correspond to adding approximately 5 min of brisk walking, 15 min of slow walking, or around 500 steps per day. In contrast, IG reflects activity intensity distribution throughout the day, without relying on traditional cut-points. IG is always negative because time accumulated decreases as intensity increases. A steeper (more negative) gradient indicates that activity is accumulated predominantly at lower intensities, with relatively little time spent in higher-intensity movement. By contrast, a shallower (less negative) gradient indicates that a greater proportion of daily activity is accumulated at relatively higher intensities (Figure 1). In addition, MX metrics (including M5, M10, M15, M30, M45, M60, and M120) were derived to characterize the acceleration levels achieved during the most active periods of the day. MX represents the minimum acceleration above which an individual’s most active X minutes are accumulated, with higher MX values indicating a greater ability to maintain higher acceleration levels during peak activity periods. Together, AvAcc, IG, and MX capture complementary dimensions of daily PA, encompassing activity volume, activity intensity distribution, and peak activity intensity.

### 2.4. Assessment of Dietary Intake and Calculation of the Diet Quality Score

Dietary intake was assessed using a validated semi-quantitative food frequency questionnaire (Table A1, Appendix A). Participants reported their consumption frequency (from “never” to “multiple times per day”) and usual portion size of each food item over the past year. Intake frequencies were converted into daily equivalents (servings/day), and daily intakes (g/day) were calculated by multiplying frequency by portion size. To reduce the influence of extreme values and potential reporting errors, dietary intake variables were winsorized at the 1st and 99th percentiles.

Diet quality was evaluated using the China Prime Diet Quality Score (CPDQS), developed by the National Institute for Nutrition and Health, Chinese Center for Disease Control and Prevention [19]. The CPDQS consists of 22 components, including dark green vegetables, dark red/orange vegetables, other vegetables, dark yellow fruits, citrus fruits, other fruits, whole grains/mixed beans, sweet potatoes, other tubers, soybeans, nuts, poultry, fish and shrimp, dairy products, eggs, livestock meat, fried foods, refined grains, sugar-sweetened beverages, salt, cooking oil, and alcohol. Scoring was based on the recommended intake amounts in the Chinese Balanced Dietary Pagoda at an energy intake level of 2000 kcal/day. Each component was assigned a score ranging from 0 to 4, and the total CPDQS ranged from 0 to 100, with higher scores indicating better adherence to dietary recommendations. Higher scores were assigned for higher intakes of recommended foods, whereas reverse scoring was applied for foods to be limited (e.g., fried foods, refined grains, sugar-sweetened beverages, salt, cooking oil, and alcohol). To reflect local rural dietary habits, several study-specific adaptations were made when mapping questionnaire items to CPDQS components. Specifically, soy intake was estimated from soy products and converted to dry soybean equivalents, “dark-colored fruits” were used instead of the original “dark-yellow fruits” category, and alcohol intake in the later questionnaire version was assessed mainly through baijiu consumption, as most rural older adults in this population consumed baijiu rather than beer or wine. Detailed food-group mapping and scoring information are provided in the Table A2, Appendix A.

### 2.5. Measurement of Covariates

**Figure A1:**
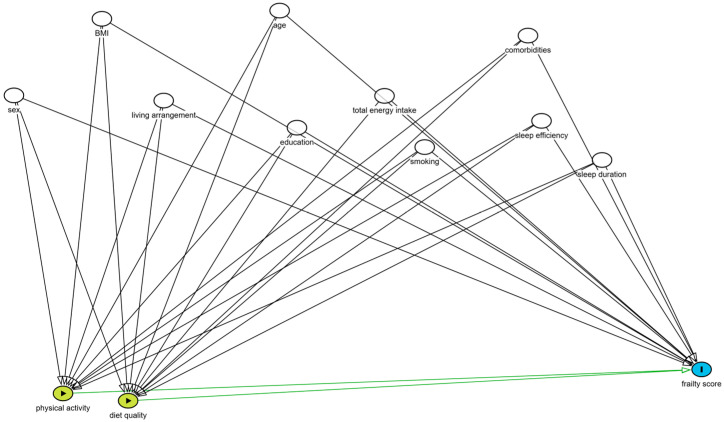
Directed acyclic graph (DAG) illustrating the hypothesized relationships between physical activity, diet quality, and frailty. Notes: Physical activity (including activity volume and intensity distribution) and diet quality were considered the main exposures, and frailty score was the outcome. The green nodes indicate the exposures, the blue node indicates the outcome, and the white nodes indicate potential confounders. Green arrows represent the primary exposure–outcome relationships of interest, whereas black arrows represent hypothesized confounding pathways. Covariates such as age, sex, education, living alone, BMI, smoking status, chronic disease status, sleep duration, sleep efficiency, and total energy intake were included as potential confounders, as they may influence both exposures and frailty.

Data on covariates were collected through one-to-one face-to-face interviews using a structured questionnaire. Potential confounders were identified based on expert knowledge and the existing literature [20,21] and were incorporated into a directed acyclic graph (DAG). The DAG was constructed using the DAGitty web-based platform (version 3.1) (Figure A1). Based on the DAG and substantive knowledge, the final multivariable models adjusted for age, sex, education level, living alone, BMI, smoking status, chronic disease status, sleep duration, sleep efficiency, and total energy intake.

Age, sex, smoking status, living alone, education level, and chronic disease status were obtained from the questionnaire. Total energy intake was estimated from the dietary assessment and expressed as kcal/day. Sleep duration and sleep efficiency were objectively assessed using a triaxial accelerometer and processed with the GGIR package. Sleep efficiency was defined as the percentage of sleep within the sleep period time window, averaged across valid days.

### 2.6. Statistical Analysis

Descriptive characteristics were summarized according to variable type and distribution. Continuous variables were presented as mean ± standard deviation (SD), and categorical variables as *n* (%). CPDQS, AvAcc, and IG were standardized as Z-scores to facilitate comparison of effect sizes. Multivariable linear regression models were used to evaluate the associations of diet quality and physical activity metrics with frailty scores, with robust standard errors applied to account for potential heteroscedasticity. Three progressively adjusted models were constructed. Model 1 adjusted for age and sex. Model 2 was further adjusted for BMI, smoking status, living alone, education level, chronic disease status, sleep duration, and sleep efficiency. Model 3 was additionally adjusted for total caloric intake (kcal/day) to account for potential confounding by overall energy intake. Analyses were conducted in three stages: (1) separate models assessing CPDQS, AvAcc, and IG individually; (2) a mutually adjusted model including all three exposures simultaneously; and (3) a joint-association analysis based on categorical combinations of diet quality and IG. For the joint-association analysis, CPDQS and IG were dichotomized into low and high groups using median splits in the main analytical sample. Multicollinearity among independent variables was assessed using the variance inflation factor (VIF), with VIF < 5 considered indicative of no substantial collinearity. Restricted cubic spline (RCS) analyses based on Model 3 were conducted to assess potential non-linear dose–response associations between lifestyle exposures and frailty scores. To assess the robustness of the findings, we conducted two sensitivity analyses: (1) excluding participants with three or more chronic conditions, and (2) excluding participants in the lowest quintile of the EuroQol visual analogue scale (EQ-VAS), a measure of self-rated health-related quality of life ranging from 0 to 100, with higher scores indicating better perceived health status. All analyses were performed using Stata 17.0 and R 4.2.2. A two-sided *p* < 0.05 was considered statistically significant.

## 3. Results

As shown in Table 1, a total of 1203 participants were included (mean age 72.49 ± 4.17 years), of whom 43.64% were male, 19.37% lived alone, and 23.52% reported no chronic diseases. The mean BMI was 24.56 ± 3.58 kg/m^2^. Most participants had primary school education or below (60.52%), and 62.84% were never smokers. The mean CPDQS was 63.08 ± 8.84; IG was −2.77 ± 0.23, AvAcc was 23.9 ± 7.93 mg, and the frailty score was 0.61 ± 0.79.

**Figure 2:**
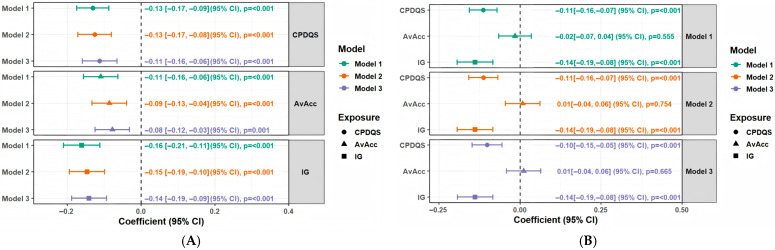
Associations of CPDQS, AvAcc, and IG (all Z-scores) with frailty score. Notes: Panel (**A**) Separate models; Panel (**B**) Mutually adjusted models. Multivariable linear regression with robust standard errors was used. Model 1 adjusted for age and sex; Model 2 additionally adjusted for BMI, smoking status, living alone, education level, chronic disease status, sleep duration, and sleep efficiency; Model 3 further adjusted for total energy intake. The dotted line represents the null value (β = 0). Results are presented as β (95% CI) with *p* values.

**Figure A2:**
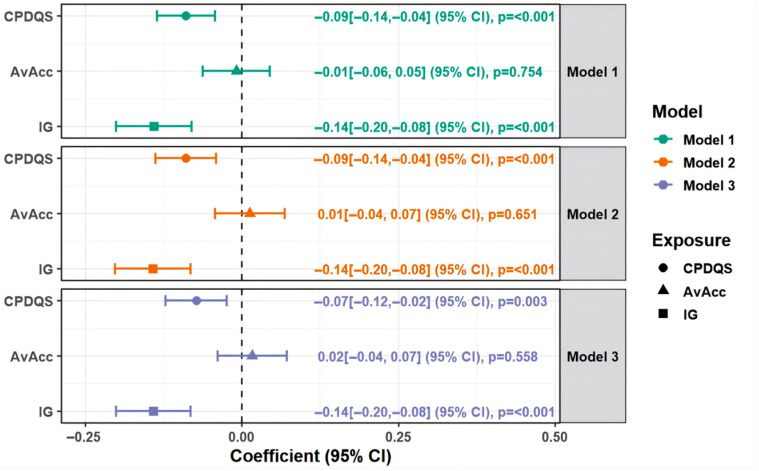
Associations of CPDQS, AvAcc, and IG (all Z-scores) with frailty score after excluding participants with three or more chronic conditions. Notes: Multivariable linear regression with robust standard errors was used. Model 1 adjusted for age and sex; Model 2 additionally adjusted for BMI, smoking status, living alone, education level, chronic disease status, sleep duration, and sleep efficiency; Model 3 further adjusted for total energy intake. The dotted line represents the null value (β = 0). Results are presented as β (95% CI) with *p* values. *n* = 948.

In the separate models, higher CPDQS, AvAcc, and IG (per SD increase) were consistently associated with lower frailty scores across progressively adjusted models (Figure 2A). However, in the mutually adjusted models including CPDQS, AvAcc, and IG simultaneously, only CPDQS and IG remained independently associated with frailty score, whereas AvAcc was not significantly associated. Specifically, in Model 3, CPDQS (β = −0.10, 95% CI −0.15 to −0.05; *p* < 0.001) and IG (β = −0.14, 95% CI −0.19 to −0.08; *p* < 0.001) were inversely associated with frailty score, while AvAcc showed no significant association (β = 0.01, 95% CI −0.04 to 0.06; *p* = 0.665) (Figure 2B). Sensitivity analyses for the multiple linear regression models showed results fully consistent with the main analysis (Figure A2 and Figure A4). In both analyses, CPDQS and IG remained significantly and inversely associated with frailty score, whereas AvAcc remained non-significant, further supporting the robustness of the main findings.

**Figure 3:**
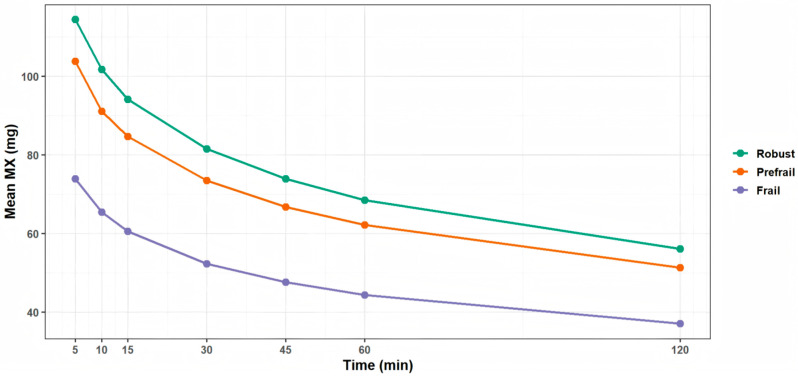
Trends in mean MX (mg) across time windows (M5–M120) by frailty status.

Figure 3 shows the mean MX (mg) values across different time windows (5, 10, 15, 30, 45, 60, and 120 min) stratified by frailty status (robust, pre-frail, and frail). Mean MX values decreased progressively as the time window increased in all groups. Across all time windows, the robust group consistently exhibited the highest MX values, followed by the pre-frail group, while the frail group showed the lowest values. These descriptive patterns indicate that greater frailty is accompanied by lower acceleration levels during the most active minutes of the day.

**Figure 4:**
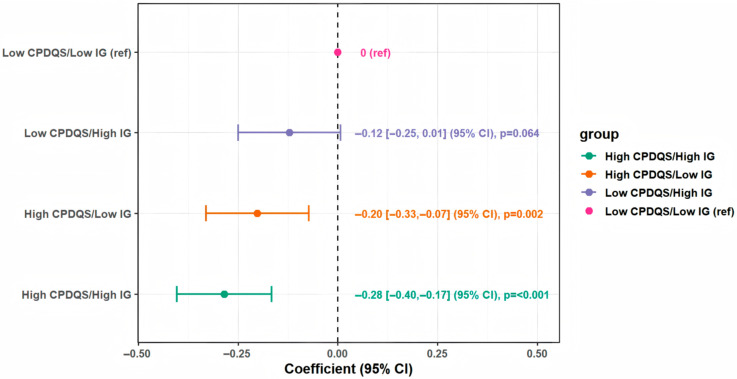
Joint association of CPDQS and IG (Z-scores) with frailty score. Notes: Models were fully adjusted for age, sex, BMI, smoking status, living alone, education level, chronic disease status, sleep duration, sleep efficiency, and total energy intake. CPDQS and IG were dichotomized into low and high groups using median splits in the main analytical sample. The dotted line represents the null value (β = 0). Results are presented as β (95% CI) with *p* values.

**Figure A3:**
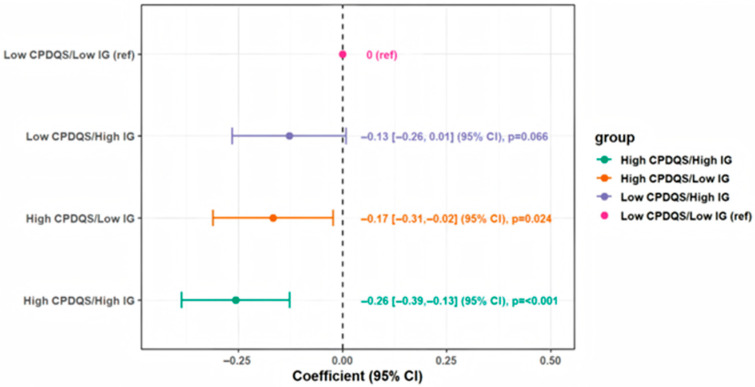
Joint association of CPDQS and IG (Z-scores) with frailty score after excluding participants with three or more chronic conditions. Notes: Models were fully adjusted for age, sex, BMI, smoking status, living alone, education level, chronic disease status, sleep duration, sleep efficiency, and total energy intake. CPDQS and IG were dichotomized into low and high groups using median splits in the main analytical sample. The dotted line represents the null value (β = 0). Results are presented as β (95% CI) with *p* values. *n* = 948.

**Figure A4:**
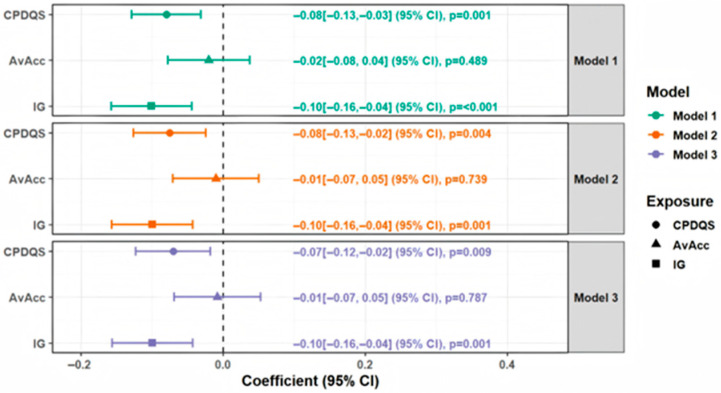
Associations of CPDQS, AvAcc, and IG (all Z-scores) with frailty score after excluding participants in the lowest quintile of the EQ-VAS. Notes: Multivariable linear regression with robust standard errors was used. Model 1 adjusted for age and sex; Model 2 additionally adjusted for BMI, smoking status, living alone, education level, chronic disease status, sleep duration, and sleep efficiency; Model 3 further adjusted for total energy intake. The dotted line represents the null value (β = 0). Results are presented as β (95% CI) with *p* values. *n* = 862.

**Figure A5:**
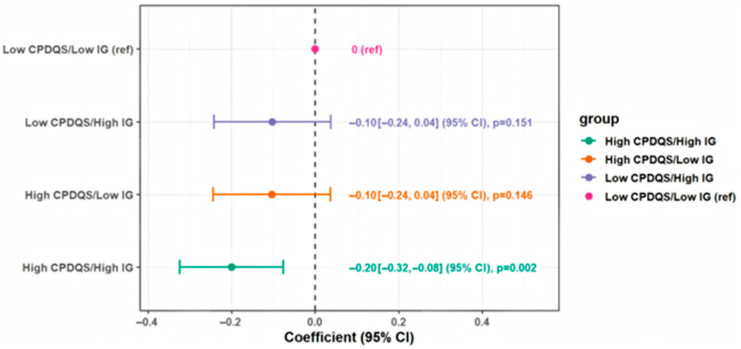
Joint association of CPDQS and IG (Z-scores) with frailty score after excluding participants in the lowest quintile of the EQ-VAS. Notes: Models were fully adjusted for age, sex, BMI, smoking status, living alone, education level, chronic disease status, sleep duration, sleep efficiency, and total energy intake. CPDQS and IG were dichotomized into low and high groups using median splits in the main analytical sample. The dotted line represents the null value (β = 0). Results are presented as β (95% CI) with *p* values. *n* = 862.

Figure 4 presents the joint association of CPDQS and IG with frailty score. Using the low CPDQS/low IG group as the reference, the high CPDQS/high IG group had the lowest frailty score (β = −0.28, 95% CI −0.40 to −0.17; *p* < 0.001), followed by the high CPDQS/low IG group (β = −0.20, 95% CI −0.33 to −0.07; *p* = 0.002). Sensitivity analyses yielded broadly similar results (Figure A3 and Figure A5). In both analyses, the high CPDQS/high IG group remained significantly associated with lower frailty scores, supporting the robustness of the main findings.

**Figure 5:**
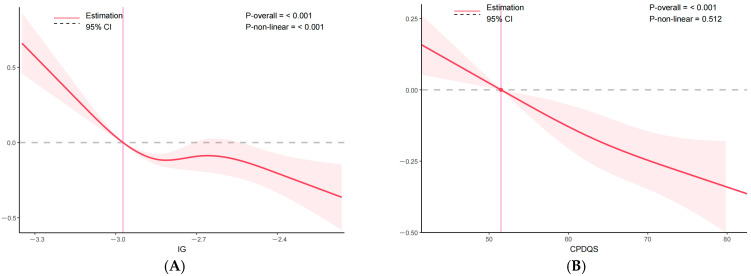
Restricted cubic spline analyses of the associations between IG and CPDQS with frailty score. Notes: Panel (**A**) IG; Panel (**B**) CPDQS. The red solid line represents the estimated association, the shaded light red area represents the 95% confidence interval, the grey dashed horizontal line indicates the null value (β = 0), and the pink vertical line indicates the reference value at which the estimated effect is set to zero. Models were fully adjusted for age, sex, BMI, smoking status, living alone, education level, chronic disease status, sleep duration, sleep efficiency, and total energy intake.

Restricted cubic spline models (Figure 5) were used to examine potential non-linear dose–response associations between exposures and frailty score. For IG, the association was statistically significant and strongly non-linear (*p*-overall < 0.001; *p*-non-linear < 0.001), showing a steep decrease in frailty score at lower IG levels followed by a plateau at higher levels. In contrast, CPDQS demonstrated an overall inverse association with frailty score (*p*-overall < 0.001) without evidence of non-linearity (*p*-non-linear = 0.512), indicating an approximately linear relationship.

## 4. Discussion

Our findings showed that IG was significantly and inversely associated with frailty, whereas AvAcc was not significantly associated with frailty. Diet quality was also independently and linearly inversely associated with frailty. These results suggest that, in this population, the activity intensity distribution may be more relevant to frailty than activity volume alone. In the joint analysis, the high CPDQS/high IG group had the lowest frailty score, and the high CPDQS/low IG group was also significantly associated with lower frailty score in the main analysis, whereas the low CPDQS/high IG group did not show a consistent association across analyses. This pattern suggests that better diet quality may have a relatively more stable association with lower frailty, while the potential benefit of a more favorable activity intensity distribution may be more evident in the context of higher diet quality.

These findings provide support for the “rural frailty paradox”, as the absence of an association between AvAcc and frailty contrasts with the prevailing assumption that higher levels of physical activity are invariably beneficial [8,22]. One possible explanation is that, unlike urban residents whose activity volume may include a greater proportion of purposeful exercise, physical activity in rural older adults is often largely derived from repetitive, low-intensity labor [23], which may be less strongly related to lower frailty than a more favorable activity intensity distribution. Unlike prior studies relying on MVPA cut-points, we adopted a cut-point-free approach. IG reduces structural biases inherent to threshold-based metrics by capturing the full intensity distribution of daily movement, thereby enabling the identification of meaningful differences in frailty levels among individuals with similar total activity volumes but distinct activity intensity distributions [12]. Collectively, these results suggest that, among rural older adults, spending more time in relatively higher-intensity activities rather than only accumulating low-intensity activity may be more strongly associated with lower frailty levels.

Our findings reveal a significant linear inverse association between CPDQS and frailty, supporting diet quality as an important lifestyle factor associated with frailty. This is consistent with previous evidence suggesting that healthier dietary patterns are associated with lower frailty risk. In particular, a recent meta-analysis including 89,860 individuals reported a dose–response inverse association between healthy dietary patterns and frailty risk [24]. Compared with studies that examined diet or physical activity separately, our joint analysis provides additional evidence that diet quality and activity intensity distribution may be jointly relevant to frailty. Notably, however, the observed pattern did not indicate that a higher IG was consistently associated with lower frailty regardless of diet quality. Rather, the potentially favorable association of IG appeared to be more evident in the context of better diet quality. In line with this, a study involving 273 older adults found that the combination of an anti-inflammatory diet and adequate physical activity was associated with lower frailty incidence [25]. Longitudinal analyses suggest that both vigorous PA and high protein intake are associated with a reduced risk of frailty, highlighting the importance of combined lifestyle factors [26].

From a biological perspective, higher-intensity activity may provide mechanical stimuli relevant to skeletal muscle adaptation and remodeling, including increased capillary density, a shift in muscle fiber phenotype from glycolytic toward more oxidative fibers (type I and IIa), and enhanced mitochondrial function [27,28]. Similarly, high-quality diets may provide biochemical substrates and a nutritional milieu supportive of these adaptations, such as indispensable amino acids for muscle protein synthesis [29] and nutrients supporting metabolic homeostasis [30]. However, these interpretations remain speculative, and further longitudinal and intervention studies are needed to determine whether combined approaches addressing both activity intensity and diet quality may be relevant to frailty prevention in rural older adults.

From a clinical and public health perspective, although no universally accepted clinically meaningful threshold for IG has been established, a higher IG may pragmatically reflect a movement pattern characterized more by activity intensity distribution than by total activity volume. For rural older adults, this suggests that frailty-prevention strategies should not only promote a healthy diet but also encourage safely increasing the intensity of everyday movements—such as engaging in brisk walking instead of slow walking—rather than merely focusing on increasing total activity volume. Low-cost technologies may also help monitor movement patterns and support individualized lifestyle guidance, although their effectiveness requires further evaluation.

This study’s strengths include the use of triaxial accelerometry and the IG/AvAcc framework, which minimized self-report bias and effectively disentangled activity volume from activity intensity distribution. However, the cross-sectional design precludes causal inference. In addition, although the dietary assessment tool was culturally adapted, dietary data may still be subject to recall bias and seasonal variation. Several issues related to CPDQS operationalization should be considered when interpreting our findings. Although the dietary assessment was adapted to local eating habits, these modifications may have reduced comparability with the original CPDQS. Specifically, soy intake was estimated from soy products and converted to dry soybean equivalents, “dark-colored fruits” were used instead of the original “dark-yellow fruits” category, and alcohol intake in the later questionnaire version was assessed mainly through baijiu consumption. These adaptations may have introduced some degree of non-differential misclassification and likely attenuated the observed associations. Therefore, caution is needed when comparing our findings with studies using the original CPDQS. In addition, valid accelerometer data were defined as ≥3 days with ≥16 h/day of wear time. This relatively lenient criterion may not fully capture habitual activity patterns. Recruitment from a single region further limits the generalizability of these findings to other rural Chinese populations.

## 5. Conclusions

In rural older adults, activity intensity distribution—but not activity volume—was independently associated with frailty. Diet quality was also inversely associated with frailty, and the most favorable frailty profile was observed among participants with both higher IG and higher CPDQS.

## Figures and Tables

**Table 1:** Baseline characteristics of the study participants (*N* = 1203).

Characteristics	Values
Age (years)	72.49 ± 4.17
Male, *n* (%)	525 (43.64)
Living alone, *n* (%)	233 (19.37)
BMI (kg/m^2^)	24.56 ± 3.58
Education level, *n* (%)	
Primary school or below	728 (60.52)
Junior high school	231 (19.20)
Senior high or vocational school	190 (15.79)
College, university or higher	54 (4.49)
Smoking status, *n* (%)	
Never smoker	756 (62.84)
Former smoker	236 (19.62)
Current smoker	211 (17.54)
Sleep duration (min/day)	326.69 ± 67.54
Sleep efficiency (ratio)	0.79 ± 0.09
Total energy intake (kcal/day)	1947.87 ± 766.86
No chronic diseases, *n* (%)	283 (23.52)
CPDQS	63.08 ± 8.84
IG	−2.77 ± 0.23
AvAcc (mg)	23.9 ± 7.93
EQ-VAS score	79.79 ± 16.02
Frailty score	0.61 ± 0.79

Notes: Values are expressed as mean ± standard deviation for continuous variables and frequency (*n*) and percentage (%) for categorical variables. Abbreviations: BMI, body mass index; EQ-VAS, EuroQol visual analogue scale; CPDQS, China Prime Diet Quality Score; IG, intensity gradient; AvAcc, average acceleration.

## Data Availability

The data that support the findings of this study are available from the corresponding author upon reasonable request.

## Abbreviations

The following abbreviations are used in this manuscript:

MVPA	Moderate-to-Vigorous Physical Activity
AvAcc	Average Acceleration
CPDQS	China Prime Diet Quality Score
IG	Intensity Gradient
EQ-VAS	EuroQol visual analogue scale
PA	Physical activity
BMI	Body Mass Index

## Appendix A

**Table A1:** Mapping food frequency questionnaire items to CPDQS components and study-specific adaptations.

Category	Sub-Category	Detailed Description
1. Staple Foods	a. Rice	White rice, rice porridge, fried rice, etc.
	b. Wheat Products	Steamed buns (Mantou), noodles, stuffed buns (Baozi), flatbreads, etc.
	c. Coarse Grains	Millet, corn, sorghum, oats, etc.
	d. Sweet Potato	Sweet potato
	e. Other Tubers	Potato, Chinese yam, taro, etc.
2. Animal-Based Foods	a. Livestock Meat and Products	Pork, beef, mutton and their processed products.
	b. Poultry and Products	Chicken, duck, goose and their processed products.
	c. Aquatic/Seafood Products	Fish, shrimp, crab, shellfish, etc. (fresh or frozen).
	d. Eggs	Chicken eggs, duck eggs, etc.
3. Dairy Products	a. Whole Milk/Yogurt	
	b. Whole Milk Powder	
	c. Skim Milk/Yogurt	
	d. Skim Milk Powder	
4. Legumes	a. Dry Beans	Soybeans, adzuki beans, mung beans, green beans, black beans, etc.
	b. Bean Products	Firm tofu, soft tofu, tofu skin, dried tofu sticks, tofu pudding, etc., excluding soy milk and other soy-based beverages.
	c. Soy Milk	
5. Vegetables	a. Total Vegetables	leafy greens (e.g., bok choy), spinach, water spinach, tomatoes, green peppers, carrots, Chinese cabbage, radish, cabbage, etc., excluding potatoes, Chinese yam, and other tubers.
	b. Dark Green Vegetables	Such as spinach, Chinese flowering cabbage (youcai), garland chrysanthemum, and lettuce, etc.
	c. Red/Yellow/Orange Vegetables	Tomatoes, bell peppers, carrots, red-hearted radish, etc.
6. Fruits	a. Total Fruits	Apples, bananas, oranges, pears, etc.
	b. Dark Fruits	Brightly colored fruits (e.g., red, orange, yellow, blue, and purple), such as oranges/mandarins, mangoes, pineapples, red-fleshed dragon fruit, mulberries, blueberries, strawberries, and cherries.
	c. Citrus Fruits	Oranges, pomelos, tangerines, grapefruits, etc.
7. Nuts		Melon seeds, peanuts, walnuts, etc.
8. Fried Foods		Deep-fried dough sticks (Youtiao), fried pancakes, French fries, potato chips, fried chicken wings, fried fish, fried dough twists (Mahua), etc.
9. Sugar-Sweetened Beverages		Cola, Sprite, iced black tea, etc.
10. Salt		Cooking salt.
11. Cooking Oil	a. Vegetable Oil	Peanut oil, corn oil, etc.
	b. Animal Fat	Lard, tallow, etc.

**Table A2:** CPDQS scoring components and cut-off values used in this study.

Positively Scored Food Components (0–4 Points)					
Food Group	0 Points	1 Point	2 Points	3 Points	4 Points
Red/Yellow/Orange Vegetables	0	(0, 20)	[20, 40)	[40, 60)	≥60
Other vegetables	0	(0, 40)	[40, 80)	[80, 120)	≥120
Dark fruits	0	(0, 20)	[20, 40)	[40, 60)	≥60
Citrus fruits	0	(0, 20)	[20, 40)	[40, 60)	≥60
Other fruits	0	(0, 20)	[20, 40)	[40, 60)	≥60
Nuts	0	(0, 4)	[4, 8)	[8, 12)	≥12
Poultry and Products	0	(0, 10)	[10, 20)	[20, 30)	≥30
Dairy Products	0	(0, 60)	[60, 120)	[120, 180)	≥180
Eggs	0	(0, 10)	[10, 20)	[20, 30)	≥30
Positively scored food components (0–8 points)					
Food Group	0 Points	2 Points	4 Points	6 Points	8 Points
Dark green vegetables	0	(0, 20)	[20, 40)	[40, 60)	≥60
Bean Products/Soy Milk	0	(0, 4)	[4, 8)	[8, 12)	≥12
Aquatic/Seafood Products	0	(0, 10)	[10, 20)	[20, 30)	≥30
Coarse Grains/Dry Beans	0	(0, 10)	[10, 20)	[20, 30)	≥30
Tubers (0–2 points)					
Food Group	0 Points	0.5 Points	1 Point	1.5 Points	2 Points
Sweet potatoes	0	(0, 20)	[20, 40)	[40, 60)	≥60
Other tubers	0	(0, 20)	[20, 40)	[40, 60)	≥60
Reverse-scored food components					
Food Group	4 Points	3 Points	2 Points	1 Point	0 Points
Livestock Meat and Products	[0, 50]	(50, 100]	(100, 150]	(150, 200]	>200
Fried foods	[0, 50]	(50, 100]	(100, 150]	(150, 200]	>200
Rice/Wheat Products	(0, 150]	(150, 300]	(300, 450]	(450, 600]	>600 or 0
Sugar-sweetened beverages	[0, 100]	(100, 200]	(200, 300]	(300, 400]	>400
Cooking oil	[0, 25]	(25, 50]	(50, 75]	(75, 100]	>100
Salt	[0, 5]	(5, 10]	(10, 15]	(15, 20]	>20
Alcohol	[0, 15]	(15, 30]	(30, 45]	(45, 60]	>60

Notes: All intake values are expressed in grams per day (g/day) based on edible portions. Scores were assigned according to the corresponding intake range for each food group. Soybeans and related products were calculated based on dry soybean equivalent, and soy products were converted according to protein content. Dairy intake was expressed as liquid milk equivalent, and other dairy products were converted according to protein content. Alcohol intake was calculated as ethanol equivalent based on beverage-specific alcohol concentration.
